# Health-related quality of life in patients with surgically treated lumbar disc herniation: 2- and 7-year follow-up of 117 patients

**DOI:** 10.3109/17453674.2011.566136

**Published:** 2011-04-05

**Authors:** Katarina Silverplats, Bengt Lind, Björn Zoega, Klas Halldin, Martin Gellerstedt, Lena Rutberg, Helena Brisby

**Affiliations:** ^1^Department of Orthopaedics, Sahlgrenska University Hospital and Institute for Clinical Sciences, University of Gothenburg, Gothenburg; ^2^Gothenburg Spine Center, Gothenburg, Sweden; ^3^Landspitali University Hospital, Reykjavik, Iceland; ^4^University West, Trollhättan, Sweden; ^5^Department of Clinical Neuroscience and Rehabilitation, Institute of Neuroscience and Physiology, University of Gothenburg, Gothenburg, Sweden

## Abstract

**Background and purpose:**

Health-related quality of life (HRQoL) instruments have been of increasing interest for evaluation of medical treatments over the past 10–15 years. In this prospective, long-term follow-up study we investigated the influence of preoperative factors and the change in HRQoL over time after lumbar disc herniation surgery.

**Methods:**

117 patients surgically treated for lumbar disc herniation (L4-L5 or L5-S1) were evaluated with a self-completion HRQoL instrument (EQ-5D) preoperatively, after 2 years (96 patients) and after 7 years (89 patients). Baseline data (age, sex, duration of leg pain, surgical level) and degree of leg and back pain (VAS) were obtained preoperatively. The mean age was 39 (18–66) years, 54% were men, and the surgical level was L5-S1 in 58% of the patients. The change in EQ-5D score at the 2-year follow-up was analyzed by testing for correlation and by using a multiple regression model including all baseline factors (age, sex, duration of pain, degree of leg and back pain, and baseline EQ-5D score) as potential predictors.

**Results:**

85% of the patients reported improvement in EQ-5D two years after surgery and this result remained at the long-term follow-up. The mean difference (change) between the preoperative EQ-5D score and the 2-year and 7-year scores was 0.59 (p < 0.001) and 0.62 (p < 0.001), respectively. However, the HRQoL for this patient group did not reach the mean level of previously reported values for a normal population of the same age range at any of the follow-ups. The changes in EQ-5D score between the 2- and 7-year follow-ups were not statistically significant (mean change 0.03, p = 0.2). There was a correlation between baseline leg pain and the change in EQ-5D at the 2-year (r = 0.33, p = 0.002) and 7-year follow-up (r = 0.23, p = 0.04). However, when using regression analysis the only statistically significant predictor for change in EQ-5D was baseline EQ-5D score.

**Interpretation:**

Our findings suggest that HRQoL (as measured by EQ-5D) improved 2 years after lumbar disc herniation surgery, but there was no further improvement after 5 more years. Low quality of life and severe leg pain at baseline are important predictors of improvement in quality of life after lumbar disc herniation surgery.

The natural course of events after sciatic pain originating from lumbar disc herniation is most often favorable, but surgery is frequently performed in patients with persistent sciatic pain (Atlas et al. 2005, Stromqvist et al. 2008). There are many different ways to evaluate the outcome after disc herniation surgery, and traditionally the treatment effects have been studied by patient-reported pain scales (VAS), return to work, functional status, radiological/imaging outcomes, and by evaluation of complication rates (Loupasis et al. 1999, Vucetic et al. 1999, Gerszten et al. 2006, Peul et al. 2007).

In recent years, outcome based on patients' own assessments, such as satisfaction with treatment (Ronnberg et al. 2007), patients' global assessment (Hagg et al. 2002), or health-related quality of life (HRQoL) (Jansson et al. 2005, Kagaya et al. 2005, Gerszten et al. 2006, Heider et al. 2007, Veresciagina et al. 2007, Jansson et al. 2009, Stromqvist et al. 2009) have gained increasing interest in spinal surgery. Furthermore, good correlations have been shown between patients' assessments and validated objective outcome scores (Hagg et al. 2002, Ronnberg et al. 2007).

The aim of using HRQoL instruments is to measure the influence of a disorder/disease on a patient's daily life and activities; they have come to be used frequently to evaluate outcome after different types of surgery (Rampersaud et al. 2008, Rolfson et al. 2009). The most popular health status instruments are the EuroQol-5 Dimension (EQ-5D) (Dolan 1997) and the 36-Item Short-Form Health Survey (SF-36) (Ware et al. 1995), which are both patient-based questionnaires. Since these instruments are not specific for a certain condition, they allow comparisons of the effects of different treatment modalities for a specific condition and also the individual effects of different medical conditions on daily life. The EQ-5D instrument can also be used in cost-effectiveness evaluations.

The main aim of the present prospective follow-up study was to follow the postoperative development of HRQoL after lumbar disc herniation surgery. Secondary aims were to evaluate potential relationships between preoperative factors and the development of HRQoL and to investigate differences between 2-year HRQoL and 7-year HRQoL (range: 5–8 years). The primary outcome variable was change in EQ-5D.

## Patients and methods

Between September 1998 and March 2002, 117 consecutive patients surgically treated for lumbar disc herniation at a single center, a university clinic in Gothenburg, Sweden, were included in the study. The inclusion criteria were CT- or MRI-verified one-level disc herniation at L4-L5 or L5-S1 level without any other spinal diseases or previous surgery at the same level. The patients had a mean age of 39 (18–66) years. 54/117 patients (46%) were women. 49/117 patients (42%) underwent surgery at the L4-L5 level and 68/117 (58%) at the L5-S1 level. The regional ethics review board approved the study (numbers 393-96 and 577-97) and all patients gave their informed consent before inclusion.

### Surgery

A midline approach was used to dissect the paravertebral muscles down to the laminae, and the interlaminar ligaments were resected. A partial laminotomy was performed when necessary. Herniated disc material and loose fragments from the disc was removed to decompress the affected neural structures. The surgery was performed with or without a microscope according to the surgeon's preference (the use of a microscope has been reported not to influence the result (Tullberg et al. 1993). 6 spine surgeons were involved in these operations.

### Baseline data and questionnaires

1–14 days before surgery, patients filled out questionnaires with baseline data (age, sex, duration of leg pain (months)) and intensity of leg and back pain (VAS) at the preoperative visit. Health-related quality of life (HRQoL) was measured at baseline, at 2 years, and at 7 (5–8) years using the EQ-5D. The EQ-5D is a HRQoL instrument designed for self-completion by the respondents, and it is in 2 parts. The first is the EQ visual analog scale (EQ-VAS), in the range 0–100 representing “worst to best imaginable health state”. The second part is a descriptive system consisting of 5 dimensions: mobility, self-care, usual activities, pain/discomfort, and anxiety/depression. Each dimension is divided into 3 levels of severity (no problems, moderate problems, or severe problems), resulting in 243 possible health states. The results can be presented as a health profile or as a global health index (Dolan 1997). The minimum value is –0.594 and the maximum is 1.0, where negative values are considered to be a health status worse than death.

The EQ-5D was compared with the VAS scores for leg pain and back pain that were obtained at baseline and at the two follow-up visits. The EQ-5D questionnaire was mailed to the patients and brought by the patients to the outpatient clinic at the 2-year follow-up. Questionnaires for the 7-year follow-up were returned by mail. If no response was received after 2 mailed reminders the patients were reminded by telephone to complete the questionnaire.

The primary outcome variables were change in EQ-5D between baseline and follow-up at 2 years and 7 years. The secondary outcome variable was change in EQ-5D between the 2-year and the 7-year follow up. The baseline factors analyzed were age, sex, duration of pain, surgical level, degree of leg and back pain (VAS), and baseline EQ-5D score.

### Statistics

We used SPSS software version 17.0 for statistical analysis. For analysis of changes in EQ-5D score, we used the paired t-test. When comparing groups, we used the independent samples t-test (using the Welch–Satterthwaite equation). Correlations between different variables were evaluated by the nonparametric Spearmen rank correlation test. The change in EQ-5D score was also analyzed in a multiple linear regression model, including all baseline factors as potential predictors. In an explorative manner, we used the same model but excluded baseline EQ-5D as a predictor. Any p-values below 0.05 were considered to be significant. The term significant should only be interpreted in its statistical sense. Since many statistical tests were performed, all p-values should be interpreted with care.

## Results

### Baseline data

Mean VAS for back pain preoperatively was 51 (SD 22) and VAS for leg pain was 60 (SD 18). Less than 6 months' duration of leg pain preoperatively was reported by 41 of the patients, 6–12 months by 37, and more than 12 months by 39.

### EQ-5D (Table)

96/117 of the patients (82%) completed the EQ-5D questionnaire at the 2-year follow-up and 89/117 (76%) completed the EQ-5D questionnaire at the 7-year follow-up. The mean difference (change) between the preoperative EQ-5D score and the 2-year score was 0.59 (p < 0.001) and it was 0.62 (p < 0.001) between the preoperative score and the score for the long-term follow-up. 82/96 patients (85%) showed improvement in the EQ-5D score between the baseline and the 2-year follow-up, and 81/89 (91%) between baseline and long-term follow up.

**Table:** EQ-5D score according to baseline data (gender, age, surgical level, and duration of leg pain) and change in EQ-5D score between preoperative registration and the follow-up visits

	n	EQ-5D at baseline mean (SD)	n	Change after 2 years mean (SD)	n	Change after 7 (5–8) years mean (SD)
Study population	117	0.12 (0.35)	96	0.59 (0.43)	89	0.62 (0.42)
Sex						
Female	54	0.09 (0.36)	46	0.62 (0.44)	47	0.65 (0.40)
Male	63	0.14 (0.33)	50	0.57 (0.42)	42	0.58 (0.44)
Age						
0–29	22	0.13 (0.30)	18	0.57 (0.44)	18	0.62 (0.45)
30–39	50	0.06 (0.33)	39	0.63 (0.45)	36	0.59 (0.44)
40–49	22	0.11 (0.38)	19	0.63 (0.39)	19	0.68 (0.32)
50–59	18	–0.05 (0.25)	16	0.43 (0.44)	14	0.57 (0.45)
60+	5	0.20 (0.40)	4	0.77 (0.21)	2	0.94 (0.53)
Surgical level						
L4–L5	49	0.14 (0.36)	42	0.56 (0.47)	41	0.57 (0.38)
L5–S1	68	0.08 (0.33)	54	0.62 (0.39)	48	0.66 (0.44)
Duration of leg pain						
< 6 months	32	0.05 (0.36)	32	0.66 (0.45)	33	0.70 (0.40)
6–12 months	32	0.15 (0.34)	25	0.53 (0.37)	25	0.55 (0.38)
> 12 months	26	0.16 (0.35)	24	0.54 (0.50)	24	0.51 (0.47)

22/89 of the patients had a decrease in EQ-5D score, 30/89 had an unchanged score, and 37/89 had a further improvement in EQ-5D score between the 2-year follow-up and the 7-year follow-up. However, the change in EQ-5D score between these follow-ups was not significant (mean change 0.03, p = 0.2).

### EQ-5D dimensions (Figure)

Preoperatively, severe problems were reported by most patients for the dimensions pain and mobility, whereas the majority reported moderate problems for the other dimensions. At both follow-ups, the patients reported fewer problems for all dimensions. At the follow-up visits, the remaining problems that were reported were mainly for the dimension pain.

**Figure:**
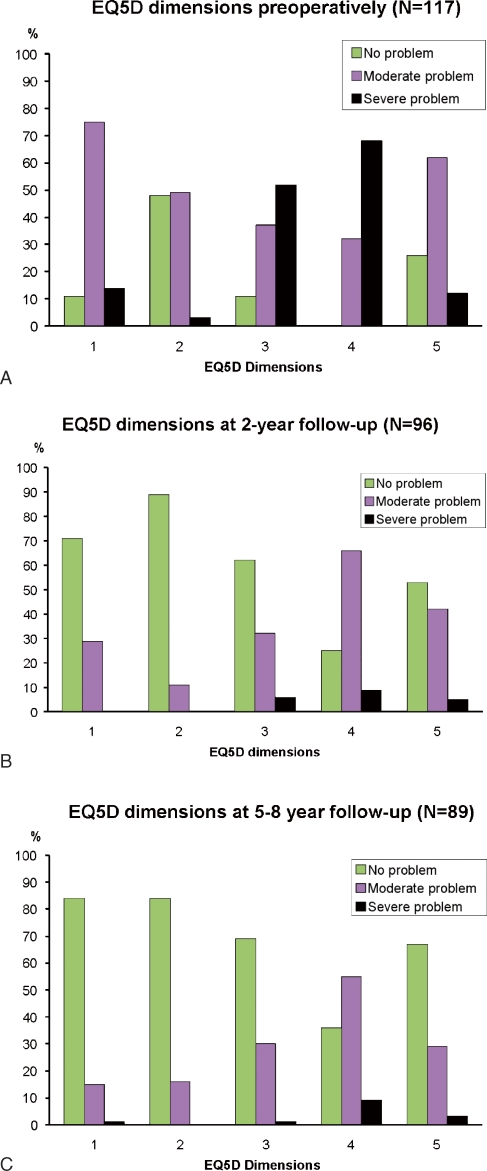
Description of the different dimensions. 1 = mobility, 2 = self-care, 3 = usual activities, 4 = pain, and 5 = anxiety/depression at baseline (panel A), at 2-year follow-up (panel B), and at long-term follow-up (panel C). There were 3 alternative answers for each dimension, according to the degree of severity: no problems, moderate problems, or severe problems.

### EQ-VAS

The mean preoperative EQ-VAS improved from 38 at baseline to 71 at the 2-year follow-up (n = 88) (p < 0.001) and from 37 to 72 at the long-term follow-up (n = 89) (p < 0.001). 76/88 patients (86%) reported a higher EQ-VAS relative to baseline after 2 years and 77/90 (86%) after 7 years. There was no significant difference in EQ-VAS between the 2-year follow-up and the 7-year follow-up.

### Baseline factors and EQ-5D

There was no significant correlation between baseline EQ-5D score and age or duration of pain; there was, however, a significant correlation between baseline EQ-5D and baseline leg pain (r = –0.40, p < 0.001) and also back pain (r = –0.34, p < 0.001). The baseline EQ-5D was not significantly different between males and females or between different operation levels.

There was no significant correlation between age and change in EQ-5D at any of the follow-ups, and there was no significant relationship between sex and change in EQ-5D.

There was no significant correlation between the duration of pain and the EQ-5D score at any time point, or in change in EQ-5D with time. We did not find any significant differences in EQ-5D score at or between time points regarding either sex or surgical level (Table).

The leg pain at baseline was correlated with the change in EQ-5D at 2-year follow-up (r = 0.33, p = 0.002) and at 7-year follow up (r = 0.23, p = 0.04). Back pain at baseline was not found to be significantly correlated with change in EQ-5D at any follow-up.

There was a correlation between baseline EQ-5D score and change in EQ-5D at both follow-ups (r = –0.70, p < 0.001 and r = –0.71, p < 0.001, respectively).

The change in EQ-5D score at the 2-year follow-up was also analyzed in a multiple regression model including all baseline factors (age, sex, duration of pain, leg and back pain, and baseline EQ-5D score) as potential predictors. The only significant predictor was found to be baseline EQ-5D score. The influence of baseline EQ-5D score was estimated (b = –1.0 (95% CI: –1.2 to -0.8)), indicating that for every unit of increase in baseline EQ-5D score, we can expect one unit less of change (improvement) in EQ-5D.

When baseline EQ-5D score was excluded from the model, the only significant predictor at the 2-year follow-up was leg pain (p = 0.04) (higher VAS leg pain score at inclusion resulted in higher change in EQ-5D). At long-term follow-up, none of the remaining predictors were significant.

## Discussion

To our knowledge, there have been no 7-year results published on EQ-5D in patients who have undergone lumbar discectomy. 82/96 (85%) of our patients who were surgically treated for lumbar disc herniation reported an improvement in HRQoL at the 2-year follow-up and 81/89 (91%) reported an improvement at the 7-year follow-up.

The mean preoperative EQ-5D score in our patients was only 0.12, which is lower than has been reported in previous studies for patients suffering from lumbar disc herniation: Jansson et al. (2005) reported a value of 0.29 and Gertzen et al. (2006) reported a value of 0.20. This may have been influenced by the long waiting time for surgery in our patients. However, there was a large and clinically relevant improvement, expressed as a change in EQ-5D score (mean 0.59), between baseline and the 2-year follow-up. Interestingly, no significant change in EQ-5D score occurred between the 2-year follow-up and the 7-year follow-up (on average, an additional change of 0.03 in EQ-5D). When looking into this more in detail, about two-fifths of the patients reported a somewhat higher EQ-5D score at the 7-year follow-up whereas only one quarter reported a lower EQ-5D score, which means that for most of the patients the result obtained after 2 years remained after 7 years.

At baseline, there was a correlation between EQ-5D and leg and back pain. The only predictors that were significantly correlated with change in EQ-5D (at both follow-ups) were leg pain at baseline and EQ-5D at baseline. In the multiple linear regression model, baseline EQ-5D score was the only significant predictor of change in EQ-5D score. Leg pain at baseline was not a significant predictor in that model, which may be explained by multicollinearity, i.e. the intercorrelation between baseline EQ-5D and leg pain. When EQ-5D was excluded from the model, leg pain was significant at the 2 year follow-up but not at the long–term follow-up.

Pain, and leg pain in particular, is the main indication for surgery in patients with disc herniation, and we found a strong correlation between EQ-5D and back pain—as well as leg pain—at baseline and at the 2-year and 7-year long-term follow-up visits. The correlation between leg and back pain was also found to be significant. Of the EQ-5D dimensions, the dimension “pain/discomfort” showed the highest frequency of “severe problems” preoperatively (68%). Compared to the study by Jansson et al (2005) (in which “severe problems” were reported by half of the patients) our study group reported having worse pain. The long waiting time for surgery at the time of the study, sociodemographic factors, and the fact that this study was performed at a university hospital with no private patients may have influenced the pain levels reported.

Even though there was a large improvement in the pain dimension at the 2-year and 7-year follow-up, 8 of 89 patients still reported having severe pain at the 7-year follow-up and as many as 64 of these 89 patients reported having moderate or severe pain.

Between half and two-thirds of the patients reported “moderate” or “severe” problems regarding mobility, self-care, anxiety, and usual activities preoperatively. However, for these 4 dimensions, more than two-thirds of the patients reported having no problems and very few reported severe problems at the follow-up visits. The postoperative values reported for these 4 dimensions at the two follow-up occasions are comparable to those of a normal population of the same age range (Burstrom et al. 2001) When comparing the EQ-5D score to a previously described age-correlated normal population, our patients had a somewhat lower EQ-5D score at both follow-ups (0.69 and 0.74 vs. around 0.8) (Burstrom et al. 2001) The somewhat lower EQ-5D score at follow-up in our patients appears to have been caused by a subgroup of patients still suffering from pain many years after surgery rather than there being an overall lower EQ5D score in the whole patient group. Back pain has been reported in up to one third of patients 5–10 years after surgery for disc herniation (Spangfort 1972, Dvorak et al. 1988). This is in agreement with our findings (20% of patients reported a back pain VAS score of > 30 at the 7-year follow-up), and it could be part of the explanation for the low EQ-5D score at the follow-up visits. The stable values of EQ-5D between the two follow-ups do not, however, suggest any deterioriation in quality of health caused by increasing back pain during the study period.

Our findings suggest that the EQ-5D instrument for disc herniation patients is highly influenced by the level of pain according to the patients' experience. Based on the fact that long-standing pain has a negative influence on most aspects of life, this result is not surprising.

Our study has several limitations, including the limited number of patients. Some acutely admitted patients may have missed being included, there was a long waiting time for surgery (about one third had leg pain more than 12 months before surgery), and there was no short-term follow-up. Comorbidity of different types, not involving the spine, may also have influenced the EQ-5D score at the follow-up visits.

In summary, in this patient population that was operated for lumbar disc herniation, health-related quality of life measured with EQ-5D was low at baseline and was strongly associated with leg pain (VAS). Quality of life improved after 2 years, with no further improvement after 5 more years. Low quality of life and severe leg pain at baseline were important predictors of improvement in quality of life after disc herniation surgery.
